# Optimizing Isolation Methods and Exploring the Therapeutic Potential of Lotus-Derived Extracellular Vesicles in Modulating Inflammation and Promoting Wound Healing

**DOI:** 10.1021/acsbiomaterials.5c00377

**Published:** 2025-06-09

**Authors:** Kai-Jiun Lo, Mu-Hui Wang, Ching-Yao Kuo, Min-Hsiung Pan

**Affiliations:** † Institute of Food Science and Technology, 33561 National Taiwan University, Taipei 10617, Taiwan; ‡ Department of Medical Research, National Taiwan University Hospital, Taipei 100225, Taiwan; § BO HUI BIOTECH CO., LTD., New Taipei City 248016, Taiwan; ∥ Department of Medical Research, China Medical University Hospital, China Medical University, Taichung 40402, Taiwan

**Keywords:** *Nelumbo nucifera*, lotus-derived extracellular vesicles, tangential flow filtration, anti-inflammation, wound healing

## Abstract

In the past decade, with the rise of research on plant-derived extracellular vesicles (PDEVs), scientists have been continuously exploring the bioactivity of PDEVs. Many PDEVs have been shown to possess a variety of biological activities. Given that the specific characteristics of EVs are believed to be related to their source cells, PDEVs from traditional Chinese medicinal herbs hold significant potential for development. In this study, lotus ( Gaertn.) leaves were selected as the source of PDEVs, and the impact of different isolation methods on their characteristics was evaluated, while their potential biological activities were also assessed. Lotus-derived EVs (LDEVs) were isolated by using tangential flow filtration (TFF), ultracentrifugation (UC), density gradient ultracentrifugation (DGU), and size-exclusion chromatography (SEC), respectively. The mean sizes of LDEVs isolated by various methods were in the range of 130–160 nm. Although the LDEVs isolated by the TFF method had a lower zeta potential, it exhibited the highest purity, with a yield of 3.69 ± 0.43 × 10^9^ particles/g lotus leaves. Notably, LDEVs isolated by different methods all demonstrated the ability to attenuate LPS-induced inflammation in RAW264.7 cells, significantly decreasing the nitrite concentration in the culture medium. Furthermore, LDEVs also showed potential for wound healing, promoting the migration of HaCaT cells in vitro. LDEVs also demonstrated internalization by RAW264.7 and HaCaT cells. These results support the potential of LDEVs for biomedical applications while also suggesting that TFF is a promising and viable strategy for large-scale PDEV isolation.

## Graphical abstract

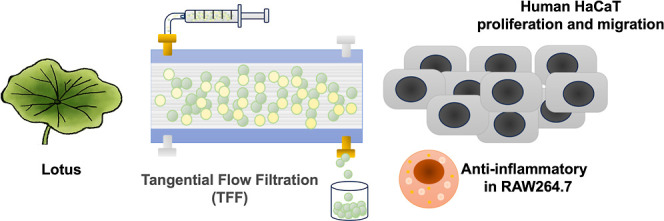

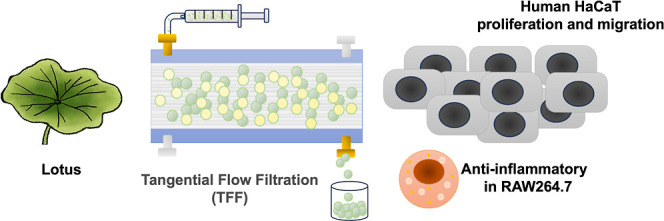

## Introduction

Extracellular vesicles (EVs) are lipid bilayer-enclosed nanoparticles secreted by various cell types across animals, plants, and microorganisms.^1–3^ They are recognized as critical mediators of intercellular communication due to their ability to transfer bioactive molecules such as proteins, lipids, and nucleic acids.^4^ Given their nanoscale size (50–1000 nm), EVs can freely traverse biological membranes,^5^ making them crucial players in numerous physiological and pathological processes.^6–8^ Recently, studies have demonstrated that plant-derived extracellular vesicles (PDEVs), similar to mammalian EVs, exhibit significant bioactivities, including anti-inflammatory, antioxidant, and microbiome-modulating properties.^9–12^ Nowadays, PDEVs have been successfully isolated from various edible plants, such as ginger,^13^ grapefruit,^14^ cabbage,^10^ etc. Moreover, PDEVs have been successfully engineered as nanocarriers for targeted drug delivery.^15,16^ These findings highlight the emerging biomedical applications of PDEVs, yet their role in skin regeneration and wound healing remains largely unexplored.

Lotus ( Gaertn.) is a widely cultivated medicinal plant with deep roots in traditional Chinese medicine. Historically, lotus leaves have been used for their anti-inflammatory, detoxifying, and circulation-improving properties.^17^ Pharmacological studies have identified flavonoids (quercetin, kaempferol), alkaloids (nuciferine), polysaccharides, and triterpenoids as key bioactive compounds responsible for antioxidant, anti-inflammatory, and tissue-regenerative effects.^18^ Notably, flavonoids and alkaloids in lotus leaves have been shown to suppress inflammatory cytokines (e.g., TNF-α, IL-6), scavenge reactive oxygen species (ROS), and promote fibroblast proliferation.^19–22^ Given these findings, lotus-derived EVs (LDEVs) may serve as bioactive nanovesicles capable of transporting and delivering these therapeutic compounds to target cells, enhancing immune regulation, and accelerating wound repair.

Like mammalian EVs, PDEVs are nanoscale and highly heterogeneous, making it difficult to isolate a pure and uniform population from plant tissues. Currently, most PDEV purification strategies are adapted from mammalian EV isolation methods,^42^ but a standardized protocol for PDEV purification has yet to be established. Traditional EV isolation method is differential ultracentrifugation (UC).^23^ However, UC requires long centrifugation times and is often impractical for large-scale applications. To overcome these limitations, researchers have explored alternative methods such as ultrafiltration,^24^ size-exclusion chromatography (SEC),^25^ polymer-based precipitation,^26^ and immuno-affinity capture.^27^ Recently, commercial EV isolation kits have been developed based on these principles, including ExoQuick (System Biosciences), Total Exosome Isolation kits (TEI, Invitrogen), qEV (Izon), exoEasy (Qiagen), ultrafiltration membrane spin columns (Millipore), and the MagCapture Exosome Isolation Kit PS (IrvineScientific). Although these kits enable faster and more user-friendly EV purification, they often suffer from small processing volumes, inconsistent purity, and batch-to-batch variability, limiting their clinical applicability.^28^ Therefore, developing a reliable and scalable purification strategy for PDEVs remains an essential step toward their therapeutic translation.

In this paper, we successfully isolated and characterized LDEVs using various purification methods and assessed their biological functions, including anti-inflammatory effects in macrophages and their wound-healing potential in keratinocytes. Our results showed that LDEVs inhibited LPS-induced inflammation and enhanced HaCaT cell viability and migration in a dose-dependent manner, significantly accelerating wound closure. These findings provide new insight into the isolation of PDEVs and highlight the potential of LDEVs to modulate inflammation and promote wound healing, supporting their therapeutic application in regenerative medicine.

## Materials and Methods

### Isolation and Purification of Lotus-Derived EVs

To isolate LDEVs, lotus leaves were used for isolation and purification of LDEVs by using UC,^12^ ultrafiltration,^29^ polymer precipitation,^10^ size exclusion chromatography (SEC),^10^ and density gradient ultracentrifugation (DGU),^12^ respectively. Briefly, lotus leaves were washed with tap water and then ground at high speed for 30 s, repeated three times, using a homogenizer. Subsequently, the juice was collected after mesh filtration, and the supernatants were collected after centrifugation at 1000*g* for 10 min, 3000*g* for 30 min, and 20,000*g* for 1 h at 4 °C. The supernatant was filtered through a 0.22 μm bottle-top vacuum filter to obtain clarified juice (S20), which was ultracentrifuged at 100,000*g* for 2 h to pellet the EVs. The EVs were washed twice with phosphate-buffered saline (PBS) and recentrifuged at the same speed. Finally, the EV pellets were suspended in PBS to obtain LDEVs (UC-LDEVs). For DGU separation, UC-LDEVs were transferred to a sucrose gradient (8%/30%/45%/60% sucrose in 20 mM Tris-Cl, pH 7.2) and centrifuged at 100,000*g* for 2 h at 4 °C. The bands between the 8–30% and 30–45% layers were collected and washed according to the above procedure, separately labeled as 8/30-LDEVs and 30/45-LDEVs. For SEC, UC-LDEVs were loaded onto SEC columns (qEV GEN2/35 nm, IZON SCIENCE LTD, New Zealand) and eluted gravitationally with PBS. Independent 1 mL fractions were collected, and those containing LDEVs (fraction 3–5 in Figure S1a) were pooled to maximize recovery and designated as SEC-LDEVs. For ultrafiltration, the tangential flow filtration (TFF) system was used with a 750 kDa molecular weight cutoff (MWCO) hollow fiber. The S20 was circulated through the system, concentrated 10-fold, diafiltered 20 times with PBS, and then finally concentrated to an appropriate volume to obtain TFF-LDEVs. For polymer precipitation, the pH value of S20 was adjusted to 5.0, mixed with PEG6000 to a final concentration of 10% (w/v), and incubated for 24 h at 4 °C. LDEVs were pelleted via centrifugation at 8000*g* for 1 h and resuspended in PBS. Finally, the sample was dialyzed for 24 h against double-distilled water using a dialysis membrane with a pore size of 100 kDa (PP-LDEVs). For the combined strategy, SEC-LDEVs were concentrated using the TFF system described above and then isolated by DGU. The bands located between the 30–45% and 45–60% sucrose gradient solution layers were collected and labeled C30/45-LDEVs and C45/60-LDEVs, respectively. All LDEVs were stored at −80 °C.

### Characterization of Lotus-Derived EVs

The size distribution and concentration of LDEVs were analyzed using a Nanoparticle Tracking Analysis Nanosight NS300 (NTA, NS300, Malvern, UK). The same parameters were used for all of the measurements. For transmission electron microscopy (TEM), LDEVs were loaded onto the Formvar carbon-coated grid for 10 min. After removing excess LDEVs, the grids were negatively stained with 2% (w/v) uranyl acetate for 1 min. Grids were dried overnight and visualized using JEM-1400 (JEOL Co., Ltd., Japan) at an accelerating voltage of 80 kV. The operation was commissioned by the Joint Center for Instruments and Researches, College of Bioresources and Agriculture at National Taiwan University. The zeta potential of LDEVs was assessed using a Zetasizer Nano ZS (Malvern).

### Anti-inflammatory Effect of LDEVs In Vitro

In order to assay the anti-inflammatory effect of LDEVs in vitro, RAW264.7 cells, obtained from ATCC, were used in this study. Briefly, 5 × 10^5^ cells were seeded into a 24-well plate (Corning Inc., USA) using Dulbecco’s modified Eagle’s medium (DMEM; Corning, USA), supplemented with 10% (v/v) fetal bovine serum (FBS; Gibco, USA). The cells were then incubated at 37 °C in a humidified atmosphere with 5% CO_2_ for 12 h. Subsequently, the phenol-red-containing medium was removed, and the cells were washed twice with PBS before the addition of phenol-red- and serum-free DMEM. Lipopolysaccharide (LPS; Sigma, USA) was concurrently added at a final concentration of 100 ng/mL to induce an inflammatory response. Ten minutes later, LDEVs were added, and the cells were further incubated for 24 h. Cell viability was measured using the MTT assay, and the nitrite assay was performed according to the Griess reaction as described previously.^30^

### Effect of LDEVs on Cell Viability of Human Keratinocyte

To assay the cell viability and proliferation, HaCaT cells were obtained from ATCC and seeded in 96-well plates (Corning Inc.) at a density of 1 × 10^4^ and 5 × 10^3^ cells/well, respectively. The cells were cultured in DMEM medium supplemented with 10% FBS and 1% (v/v) penicillin and streptomycin (Gibco, USA) and incubated at 37 °C in a humidified atmosphere with 5% CO_2_ overnight. Afterward, the culture medium was then replaced with fresh DMEM medium supplemented with 10% FBS and containing different concentrations of LDEVs. The control group received only DMEM without LDEVs. The Alamar Blue reagent was added to each well according to the manufacturer’s instructions, and absorbance was measured at 570 and 600 nm at 24, 48, and 72 h post-treatment to evaluate cell viability.

### In Vitro Wound Healing Assay

To assess the effect of Lotus-derived extracellular vesicles (LDEVs) on HaCaT cell migration, a scratch wound assay was performed using an insert-based method. HaCaT cells were seeded into 24-well plates at a density of 1 × 10^6^ cells/well in high-glucose DMEM supplemented with 10% FBS and cultured until reaching 100% confluence. A uniform cell-free area was created by removing the insert. The culture medium was then replaced with fresh DMEM supplemented with 10% FBS and treated with different concentrations of LDEVs, while the control group received culture medium without LDEVs. Cells were incubated in Ibidi Stage Top Incubator system, and cell migration was monitored using time-lapse imaging with the Leica DFC360 FX microscope (Leica Microsystems, Wetzlar, Germany), and images were captured at 0, 24, 48, and 72 h post-treatment. The wound closure area was analyzed by using ImageJ software to quantify cell migration rates.

### Labeling of LDEVs and Cellular Uptake In Vitro

LDEVs were labeled with PKH26 Fluorescent Cell Linker Kits (Sigma) in accordance with the manufacturer’s instructions. LDEV samples were suspended in 250 μL of diluent C with 2 μL of PKH26 (at a final concentration of 8 μM) and subsequently incubated for 30 min at room temperature. After centrifugation for 5 min at 12,000*g* using a centrifugal filter (30 kDa MWCO), the pellet was washed twice with PBS to remove the free PKH26 dye, finally, PKH26-labeled LDEVs were resuspended for further experiments. For in vitro uptake, cells were incubated for 4 h with 1 × 10^11^ PKH26-labeled LDEVs. After being washed twice with PBS, cells were fixed with 2% paraformaldehyde for 10 min and treated with 0.1% Triton X-100 for 10 min. Subsequently, the permeabilized cells were washed 2–3 times in PBS. 100 μL of Flash Phalloidin Green 488 (BioLegend, Inc., USA) working solution (1:50) and DAPI (1 μg/mL) were added and incubated at room temperature for 30 min. The cells were rinsed gently 2–3 times with PBS to remove excess dye. The fluorescent images were observed using fluorescence microscopy, and fluorescence intensity was assayed by CytoFLEX FLOW Cytometer (Beckman Coulter, USA).

## Results

### Characterization of Plant-Derived Extracellular Vesicles from Lotus Using Different Methods

In order to successfully isolate PDEVs from lotus leaves, we initially isolated LDEVs using various methods including TFF, UC, DGU, and polymer precipitation. These methods are commonly applied for the isolation and purification of PDEVs.^31^ As shown in Figure 1a, the particle size distribution of LDEVs isolated using different methods was similar to the majority of particles distributed between 50 and 300 nm. The average particle sizes were 159.5 ± 48.5 nm (TFF-LDEVs), 166.5 ± 64.3 nm (UC-LDEVs), 163.2 ± 70.1 nm (SEC-LDEVs), and 152.4 ± 49 nm (PP-LDEVs). Notably, LDEVs obtained from the 30–45% and 45–60% layers in DGU exhibited smaller particle sizes, approximately 132.6 ± 39.9 nm (30/45-LDEVs) and 136.3 ± 52 nm (45/60-LDEVs), respectively. Furthermore, precipitated pellets were observed at the bottom of the centrifuge tube (Figure S1b). These pellets may consist of particles that aggregated into larger clusters due to compressive forces during centrifugation, resulting in sedimentation. Although resuspending these pellets in PBS yielded an average size of approximately 153.4 ± 4.3 nm (Figure S1c), the observed aggregation during subsequent storage indicated particle instability; therefore, this sample was excluded from further experiments. It was noteworthy that the LDEVs isolated by these methods displayed several distinct peaks in their particle size distributions. Specifically, SEC-LDEVs exhibited a greater proportion of smaller particles.

**Figure 1:**
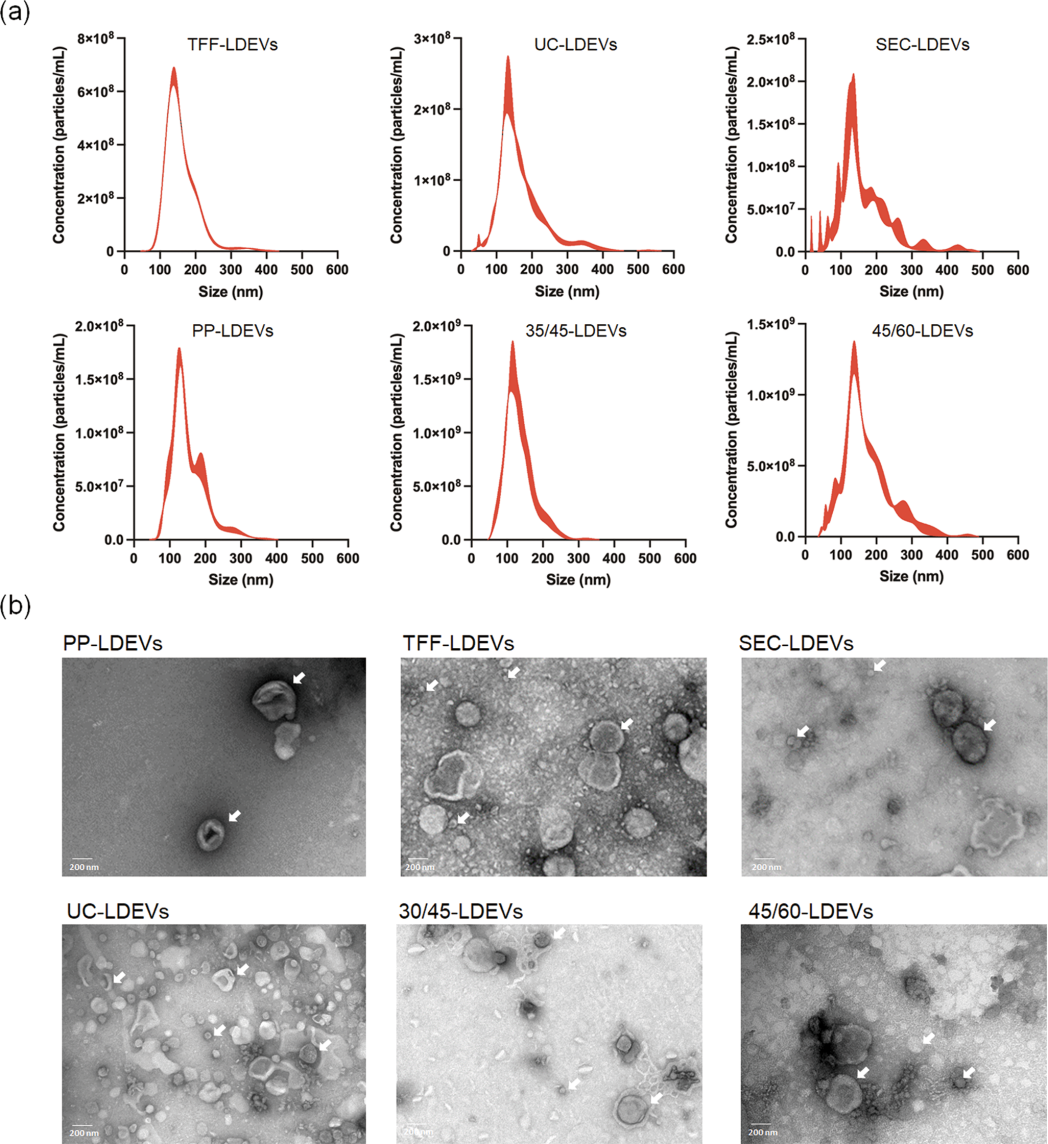
Size distribution and morphology of lotus-derived EVs (LDEVs) isolated by different methods. (a) The size distribution of LDEVs was analyzed by NTA. (b) The morphology of LDEVs was observed under TEM with negative staining. The white arrow indicates the typical cup-shaped appearance of vesicles under TEM.

On the other hand, the morphologies of LDEVs were analyzed using TEM (Figure 1b). The results revealed cup-shaped structures (indicated by the white arrows), typical characteristics of vesicles, primarily attributed to the collapse of the vesicle center during drying.^32^ Furthermore, the observed particle size was similar to those measured by nanoparticle tracking analysis (NTA), confirming that the particle size analyzed by NTA accurately represents the actual sizes of LDEVs. Moreover, the stability of the LDEVs was assessed by measuring the zeta potential. As shown in Figure 2a, the average zeta potentials of LDEVs isolated by different methods were all less −20 mV, indicating that all LDEV samples are relatively stable. Notably, the average zeta potentials of SEC-LDEVs, UC-LDEVs, DGU30-45%-LDEVs, and DGU45-60%-LDEVs were even lower than −30 mV. It was considered good and excellent stability.^33^ Additionally, we found that LDEVs contain miRNAs (Figure S2).

**Figure 2:**
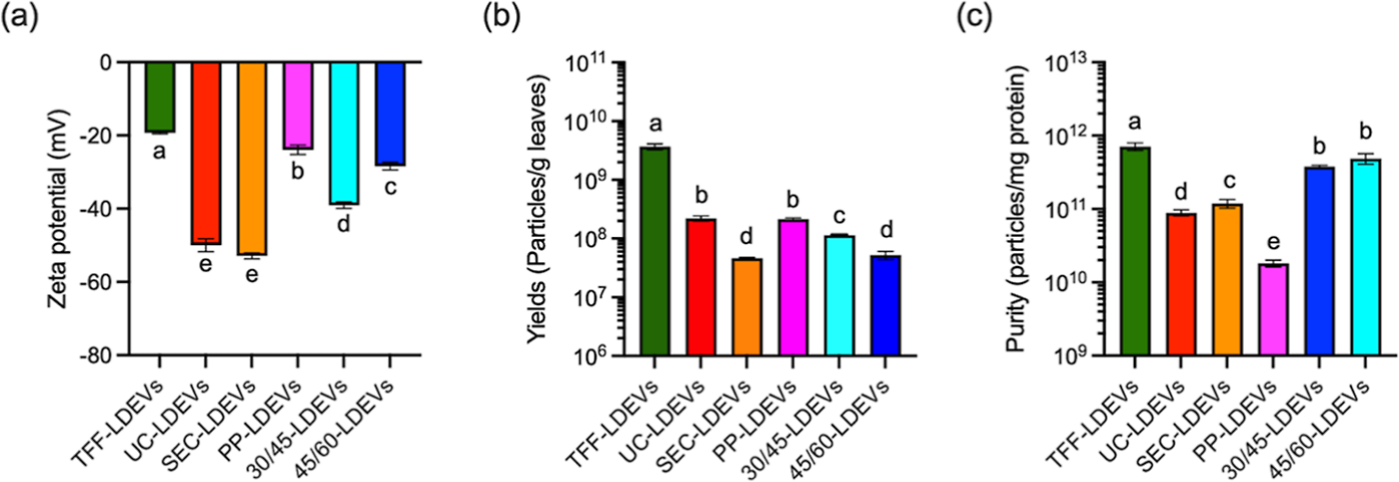
Characteristics and yields of lotus-derived extracellular vesicles (LDEVs) isolated by different methods. (a) Zeta potential of each LDEV sample analyzed by Zetasizer Nano ZS under the same conditions. (b) Quantification of LDEV yield by vesicle number. (c) Quantification of LDEV purification from LDEVs. Data are presented as means ± standard deviation, and statistical analyses were conducted using one-way ANOVA with Tukey’s post hoc analysis. The bar labeled with different letters indicates significant differences (*p* < 0.05).

### Yield and Purity of LDEVs Are Significantly Affected by the Isolation Methods

To verify the biological activity of LDEVs and evaluate their potential for future applications, it is necessary to understand the yield and purity of LDEVs obtained from different methods. Figure 2b presents the yields of LDEVs isolated by various methods. The results indicated that the highest yield was achieved with TFF-LDEVs, at approximately 3.69 ± 0.43 × 10^9^ particles/g lotus leaves, followed by UC-LDEVs and PP-LDEVs, which yielded 2.20 ± 0.22 × 10^8^ and 2.13 ± 0.12 × 10^8^ particles/g lotus leaves, respectively. In contrast, SEC-LDEVs had the lowest yield at approximately 4.63 ± 0.12 × 10^7^ particles/g of lotus leaves. Furthermore, a comparison between 30/45-LDEVs and 45/60-LDEVs revealed that the yield of 30/45-LDEVs was about twice that of 45/60-LDEVs. Since DGU serves as a further fractionation of total nanoparticles, this suggests that the majority of LDEVs have densities in the range of 1.13–1.2 g/mL, a range commonly associated with animal cell-derived exosome. Therefore, it is inferred that the LDEVs obtained from lotus leaves mostly are EVs resembling exosomes. Moreover, to assess purity, total protein content was measured in LDEV samples. The results showed that TFF-LDEVs had highest purity (7.23 ± 0.84 × 10^11^ EV particles/mg protein), while SEC-, 30/45-, and 45/60-LDEVs also exceeded 1 × 10^11^ EV particles/mg protein. In contrast, PP-LDEVs exhibited the lowest purity (about 1.71 ± 0.1 × 10^10^ EV particles/mg protein) (Figure 2c). This result is consistent with previous reports of lower purity for PDEVs isolated via PEG precipitation.^10^ This may be attributed to PEG-induced protein aggregation in the biofluid, resulting in coprecipitation, or to contamination with residual PEG.^34^ Beyond using single methods to isolate LDEVs, we explored an integrated SEC-DGU strategy to enhance LDEV purity. Figure S1c,d shows the yield and purity of LDEVs obtained using this combined method. The results indicated that there was no significant difference in purity when LDEVs were purified using the combined SEC-DGU method, with purity values all exceeding 1.0 × 10^11^ particles/mg protein (Figure S1d). It is worth noting that the yield ratio and zeta potential of C30/45- and C45/60-LDEVs are similar to those of 30/45- and 45/60-LDEVs, indicating that SEC cannot effectively separate EVs based on density and that the EVs in the SEC-LDEV sample were heterogeneous in density. In summary, the combined method did not effectively improve the purity of LDEVs; high-purity LDEVs and the highest yield were obtained by using ultrafiltration alone.

### LDEVs Inhibited the LPS-Induced Macrophage Inflammation

Since EV bioactivity is linked to the originating cell’s state and lotus leaves possess anti-inflammatory properties, we deduced that LDEVs may also have anti-inflammatory effects.^35^ To study the anti-inflammatory activities of LDEVs, RAW264.7 cells were supplemented with LDEV samples isolated from different methods and treated with LPS to induce inflammation. As shown in Figure 3a,b, when RAW264.7 cells were incubated with LPS, NO was produced due to the inflammatory response, resulting in increased nitrite levels in the culture medium. However, treatment with LDEVs significantly decreased the nitrite concentration compared with that of the LPS-only group. Furthermore, this decrease was dose-dependent, indicating that the nitrite reduction could be attributable to the effect of LDEVs. On the other hand, it was also found that LPS induction significantly decreased the viability of RAW264.7 cells (Figure 3c,d). However, when cells were cocultured with TFF-, SEC-, or PP-LDEVs, cell viability increased with increasing LDEV concentration. In particular, the cell viability of PP-LDEV treatment was comparable to the control group (Figure 3c,d). Unexpectedly, UC-, 30/45-, and 45/60-LDEVs had no significant effect on cell viability, and UC-LDEVs even decreased cell viability at a concentration of 1 × 10^11^ particles/mL. Although not significantly different compared with the LPS-induced group, TFF-, 30/45-, and 45/60-LDEVs exhibited a similar trend of reduced cell viability at high concentrations. This suggests that variations in purification methods may lead to diverse biological activities among LDEVs. In summary, regardless of their origin, LDEVs present a potential for anti-inflammatory activity. However, considering the yield and purity, TFF-LDEVs were selected for use in subsequent experiments.

**Figure 3:**
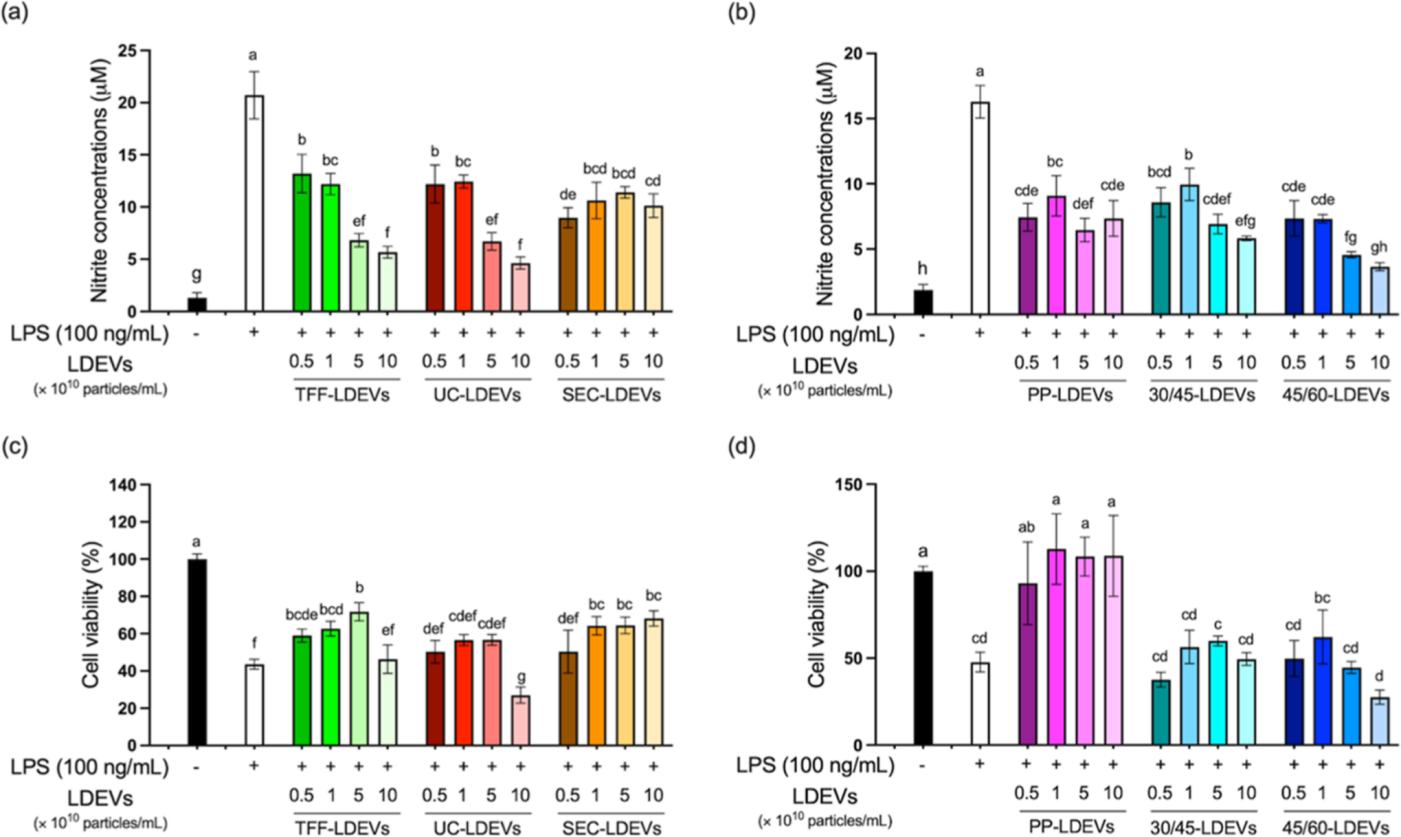
Anti-inflammatory effect of lotus-derived EVs (LDEVs) isolated using different methods. RAW264.7 macrophage cells were exposed to LPS and subsequently incubated with LDEVs isolated using TFF, UC, and SEC methods, respectively. Cell inflammation was assessed through nitrite quantification (a,b). Cell viability assays were performed using the MTT assay (c,d). Data are presented as means ± standard deviation, and statistical analyses were conducted using one-way ANOVA with Tukey’s post-hoc analysis. Bars labeled with different letters indicate significant differences (*p* value < 0.05).

### LDEVs Enhance HaCaT Cell Viability and Promote Wound Healing through Dose-Dependent Migration Stimulation

Wound healing is widely known to be a complex physiological process involving a series of responses including inflammation, cell regeneration, and cell migration. However, excessive inflammation can negatively affect this process. Therefore, appropriate anti-inflammatory strategies can help mitigate this overreaction. Given our previous findings that LDEVs possess anti-inflammatory capabilities, this study further explores the potential of LDEVs to promote wound healing. First, after adding LDEVs to the culture medium of HaCaT cells, cell viability was assessed using the Alamar Blue assay at 24, 48, and 72 h (Figure 4a). The results demonstrated that the cell viability of the 5 × 10^10^ and 1 × 10^11^ particles/mL groups was significantly higher than that of the control group at 24 h. Furthermore, after 24 h, it was found that low concentration of LDEVs (1 × 10^10^ particles/mL) treatment also significantly increased the cell viability. Noteworthy, the highest concentration of LDEVs (2 × 10^11^ particles/mL) did not exhibit significant differences in cell viability compared to the control group during the experimental period, indicating that only an appropriate concentration of LDEVs contributes to enhanced HaCaT cell viability.

**Figure 4:**
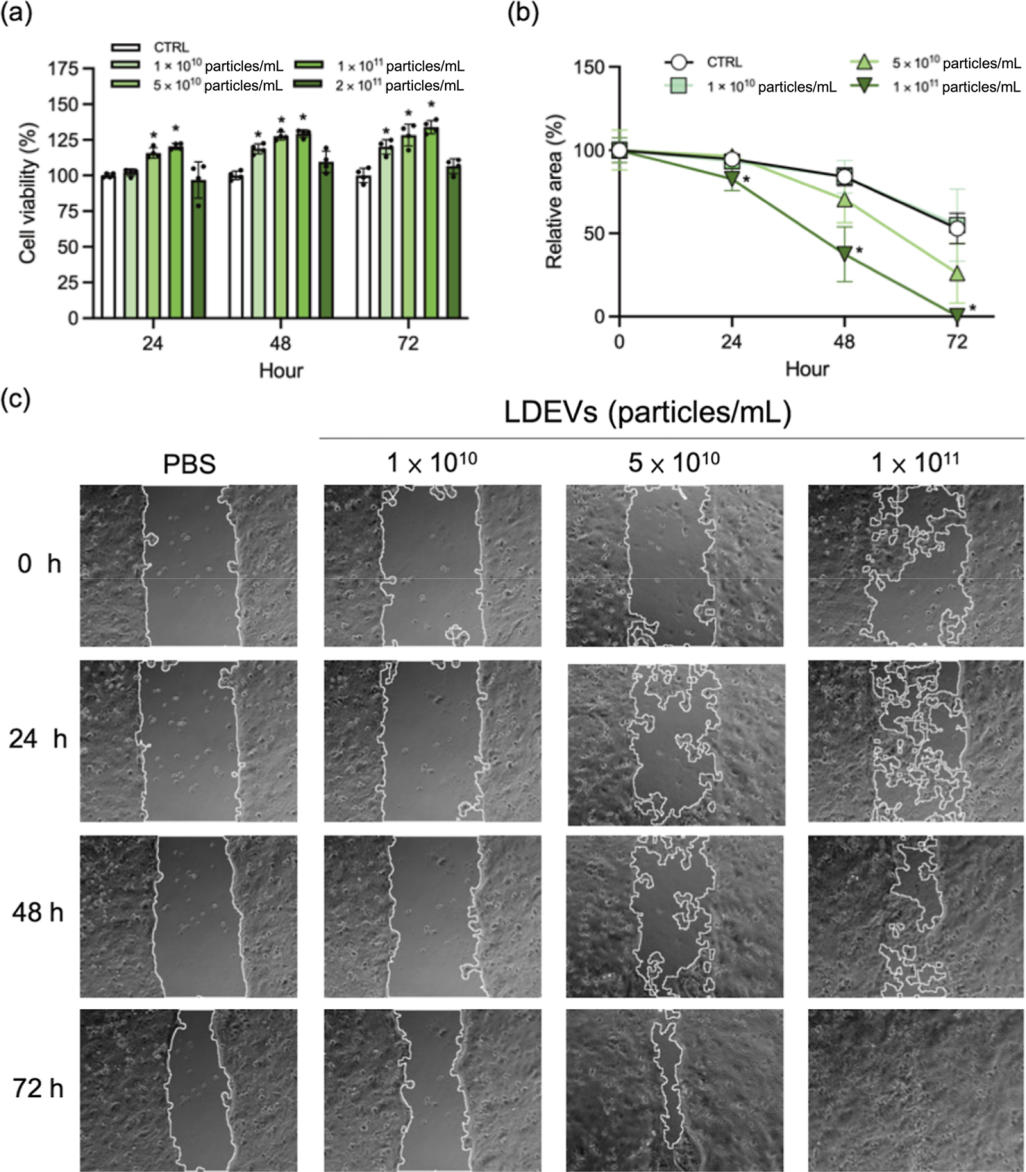
LDEVs enhanced cell viability and wound healing. HaCaT cells were incubated with LDEVs, and cell viability and wound healing closure were assessed by MTT and imageJ, respectively. (a) The cell viability of human keratinocyte (HaCaT cell) was significantly increased after coincubated with LDEVs. (b) LDEVs reduced the wound area compared with control group after 24 h. (c) Image of the wound healing assay experiment treated with different concentrations of LDEVs. Data are presented as means ± standard deviation, and statistical analyses were conducted using one-way ANOVA with Tukey’s post hoc analysis. The bar labeled with different letters indicates significant differences (*p* value < 0.05).

In addition, LDEVs promoted the HaCaT cell migration. As shown in Figure 4b,c, treatment with 5 × 10^10^ and 1 × 10^10^ particles/mL resulted in noticeable wound closure by 48 h. Statistical analysis (Figure 4b) revealed that the 1 × 10^11^ particles/mL group exhibited significantly larger wound closure areas from 24 to 72 h. These results suggest that an appropriate concentration of lotus EVs effectively promotes HaCaT cell migration, thereby accelerating wound healing.

### Internalization of LDEVs by RAW264.7 and HaCaT Cells

Given that LDEVs exhibited potential anti-inflammatory and wound-healing abilities, to further demonstrate that these bioactivities originate from LDEVs, we investigated whether LDEVs can be taken up by RAW264.7 and HaCaT cells. To evaluate the internalization of LDEVs by cells, LDEVs were labeled with PKH26 (called as LDEVs/PKH26) and coincubated with RAW264.7 and HaCaT keratinocytes, followed by fluorescence microscopy and flow cytometry analysis. As shown in Figure 5a, the PKH26 fluorescence signal (red) was observed in both RAW264.7 and HaCaT cells, indicating effective LDEV uptake. Based on the F-actin cytoskeleton (green) and nuclear counterstain (DAPI, blue), they provided structural reference points for intracellular localization. Merged images demonstrated that LDEVs/PKH26 were distributed primarily in the cytoplasm. On the other hand, flow cytometry further validated the uptake of LDEVs/PKH26 in RAW264.7 and HaCaT cells (Figure 5b,c). Compared to the control groups (unstained cells), a significant rightward shift in fluorescence intensity was observed, and the mean fluorescence intensity (MFI) also significantly increased in LDEVs/PKH26-treated cells. It suggested that both RAW264.7 and HaCaT cells exhibited significant uptake of LDEVs. Interestingly, the MFI of RAW264.7 cells was higher than that of HaCaT cells, suggesting that macrophages might exhibit a more efficient uptake mechanism, potentially due to their phagocytic activity. In summary, these data demonstrate that LDEVs can be effectively internalized by both immune and epithelial cells. Thus, we inferred that LDEVs could transport encapsulated cargo to human cells, subsequently regulating target cell biological activities. Further mechanistic studies are needed to elucidate the signaling pathways involved in LDEV-mediated cellular responses.

**Figure 5:**
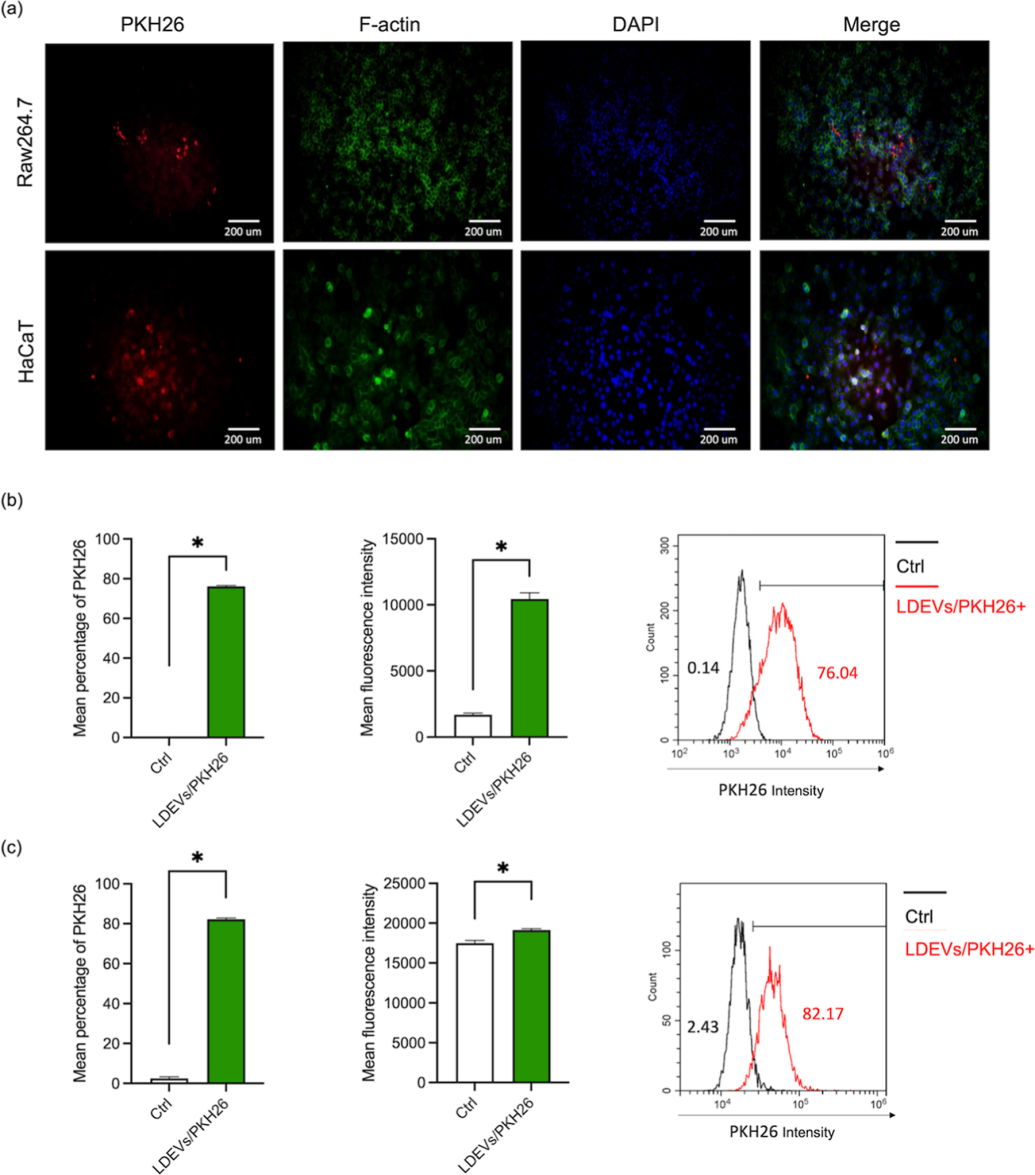
Cellular uptake of PKH26-labeled LDEVs in RAW264.7 and HaCaT cells. LDEVs were labeled with PKH26 and were incubated with cells for 4 h. (a) Schematic representation showing PKH26-labeled LDEV uptake and location within cells as visualized by fluorescence microscopy. Frequency of PKH26-labeled LDEVs in RAW264.7 (b) and HaCaT (c) cells was assessed using flow cytometry. The representative flow cytometry histograms depicting PKH26 fluorescence in cells treated with PKH26-labeled LDEVs (red line) as well as without treatment (black line). Numbers in histogram indicate the percentage of cells positive (red)/negative (black) for PKH26 fluorescence.

## Discussion

The lotus, a perennial aquatic plant , is widely cultivated in Asia and has long been used as a traditional Chinese herbal medicine. Numerous studies have confirmed that lotus leaves are rich in various phytochemicals, possessing potent antioxidant and anti-inflammatory properties.^18^ Although it is well-known that lotus leaf phytochemicals possess various bioactive functions, the extracellular vesicles present in lotus leaves are particularly poorly understood. Here, we assessed various isolation methods for lotus-derived EVs and explored their potential applications in anti-inflammatory treatment and wound healing.

Specifically, using the TFF can effectively isolate and purify LDEVs, achieving yields that are ten times higher than those obtained with other methods (Figure 2b). In addition, the isolated LDEV samples exhibited the highest purity (Figure 2c). Previous report has shown that the TFF can isolated EVs with the highest yield while avoiding damage to their structural integrity.^29^ Although SEC has been shown to further purify PDEVs,^10^ in our study, we found that the use of TFF alone was sufficient to obtain high-purity lotus leaf-derived EVs (LDEVs) (Figures 2c and S1e). This outcome is likely highly dependent on the sample type.^36,37^ Meanwhile, it may also be related to the composition of the ultrafiltration membrane chosen. For example, it has been reported that the recovery and purity of EVs using regenerated cellulose membranes is higher than that one isolated using poly­(ether sulfone) (PES) membrane. On the other hand, the particle number of EVs using the membrane with low MWCO is higher than using high one.^38,39^

Previous studies have shown that isolating EVs using the SEC column can achieve the highest purity, as it effectively removes lipoprotein contaminants and can even eliminate nonvesicular microRNA (miRNA) contamination.^37,40^ However, plants do not contain as many lipoproteins as animals, so the remaining concern is primarily non-EV miRNA, which could be co-enriched with the EVs during isolation.^41,42^ Since many studies suggest that miRNA within EVs contributes to their biological activity, eliminating non-EV miRNA during purification is crucial.^31^ Some research has demonstrated that SEC can effectively remove non-EV miRNA contamination during EV purification.^37,40,43^ However, although we did not analyze the levels of non-EV miRNA in this study, the LDEVs isolated by using TFF are not expected to be significantly affected by this issue. This is because the pore size of the membrane we used was 750 kDa MWCO. In the past, it was demonstrated that miRNA completely penetrated through a 300 kDa MWCO membrane, whereas over 50% of the miRNAs were retained in the solution when using a 100 kDa MWCO filter.^44^ It is well-known that mature miRNA loads onto Argonaute (AGO) protein (about 96 kDa), forming a complex. However, 300 kDa MWCO membranes are expected to be permeable to this complex.^44^ Several studies have shown that using a 300 kDa MWCO membrane for EV isolation can remove non-EV miRNA contamination.^45,46^ As for long non-EV mRNA, it is difficult for long RNA molecules to survive without the protection of a vesicle membrane.^47^ These results alleviate concerns regarding potential miRNA contamination of LDEVs purified by using a 750 kDa MWCO membrane.

On the other hand, using the ratio of total protein content to EV number as a measure of purity is based on assumptions made under specific conditions, and this index was initially proposed to evaluate the purity of animal-derived EVs.^36^ A significant reason for this is the high propensity of lipoproteins to be coisolated with EVs during purification.^42^ Therefore, applying this ratio of protein concentration and EV numbers to assess the purity of the EVs seems to be rational. However, plants do not contain lipoproteins in the traditional sense, so the validity of this index as a true representation of PDEV purity needs further investigation. Even though a previous study showed that the purity of PDEV extracts is associated with isolation methods, it has been reported that the protein composition of EVs varies significantly depending on the isolation method, indicating that the isolated EVs may belong to different subtypes, which could also affect protein quantification.^10,37^ Due to the lack of a specific protein biomarker, it remains unclear whether the lower purity of PDEVs results from co-isolated proteins. Therefore, it is difficult to compare the purity of EVs isolated under different conditions. However, to be cautious, we still attempted a strategy that combined methods for the further purification of LDEVs. Although this approach not only increases the time and complexity of purification but also reduces the purification efficiency, it has been reported to be effective in removing contaminants by combining two separation methods.^48–50^ Interestingly, our results showed that there was no significant change in the purity (Figure S1e). A possible reason may be the use of a 750 kDa MWCO filter membrane in the upstream process for LDEV isolation. It has been suggested that the size of small exosomes is around 30 nm due to the limitations of lipid bilayer sphere self-assembly. Although a high amount of EVs can be obtained using a small MWCO filter, non-EV protein aggregates are also collected simultaneously. To address this issue, we selected a 750 kDa MWCO filter membrane, whose pore size is approximately 30 nm, based on the Certificate of Analysis (COA) of the hollow fiber. Therefore, using a 750 kDa MWCO filter membrane for LDEV isolation is expected to achieve a better balance between yield and contaminant removal, thereby not affecting downstream purification.

Another potential reason, as mentioned above, is that the isolated LDEVs may belong to different subtypes. To date, three types of PDEVs have been identified in plants; however, it is difficult to distinguish their subtypes in the absence of specific biomarkers.^31^ Nevertheless, based on the principles of the isolation methods we used, all LDEVs, except for DGU-LDEVs, contain at least two populations of EVs with different densities. The results of the zeta potential assay also suggested the possibility of heterogeneous EVs resulting from different isolation methods (Figure 2a). Fortunately, in our study, although these LDEVs are heterogeneous and the purity of LDEVs samples differed significantly when evaluated using the ratio of protein content to EV number, the LDEVs obtained through different purification methods did not show significant differences in biological functionality. These LDEVs all exhibited the effect of mitigating LPS-induced inflammation in RAW264.7 cells (Figure 3). Similar results have been found in turmeric-derived and ginger-derived EVs, where EVs obtained from the 8:30% layer and the 30:45% layer in the DGU also exhibited bioactivity.^13,51^ A greater emphasis should perhaps be placed on analyzing the biological functionality of LDEVs obtained through various isolation techniques or on discovering suitable biomarkers. The guidelines published by the International Society for Extracellular Vesicles (ISEV) also offer similar recommendations regarding the isolation of EVs. Due to the lack of standardized isolation protocols, EV isolation should be tailored to the source and intended application in order to obtain a sufficient quantity of useable EVs.^52^ For instance, provided that contaminants do not definitively affect downstream research or applications, a slight amount of contamination could be ignored.^53^ In summary, these results re-emphasize that the use of TFF alone was sufficient to isolate LDEVs with high purity.

Another method that could be used for large-scale extraction and purification of lotus leaf exosomes is polymer precipitation. Although the results showed that the yields of PP-LDEVs are similar to UC-LDEVs, ten times lower than TFF-LDEVs (Figure 2b), and with the lowest purity (Figure 2c), it also showed a potential ability of anti-inflammatory (Figure 3b,d). Moreover, polymer precipitation method could offer several advantages. For example, it does not require specialized equipment, has a lower operational barrier, and it is accessible. Additionally, some studies indicate that PDEVs obtained by PEG precipitation retain their bioactivity and exhibit increased particle stability and uptake efficiency by animal cells.^54–56^ Therefore, when dealing with large volumes of biological samples, polymer precipitation could be another good option.

For biological functions, it has been demonstrated that lotus leaf could prevent inflammatory responses in macrophages via JNK/NF-κB signaling pathway.^35^ These results may be attributed to the abundance of phytochemicals it contains, such as polysaccharides, essential oils, flavonoids, alkaloids, and triterpenoids.^57^ These phytochemicals have all been shown to possess various biological activities, including anti-inflammatory effects.^17^ Similar to lotus leaf extract, LDEVs also exhibit anti-inflammatory capabilities (Figure 3). Although the underlying mechanism remains unclear, it is possible that this effect is due to phytochemicals encapsulated within the PDEVs, which are released into the cytoplasm after LDEV uptake by cells (Figure 5). This is plausible, as PDEVs are known to encapsulate phytochemicals.^13,51^ For example, evidence suggests that ginger-derived EVs encapsulating shogaol contribute to the induction of Nrf2 in a TLR4/TRIF-dependent manner.^13^ However, further analysis is needed to determine which specific phytochemicals are present within the LDEV cargo. Additionally, it is also possible that miRNAs within the LDEVs regulate the host cell. Previous studies have shown that plant miRNAs can regulate biological activity across species and can be delivered to target cells via PDEVs, thereby further regulating gene expression.^12^ For instance, Xu et al. demonstrated that ginseng-derived EVs containing mtr-miR159a significantly activated the phosphoinositide 3-kinase (PI3K) signaling pathway.^58^ Zheng et al. found that osa-miR166d-5p- and gma-miR396a-3p in tea-derived EVs target the 3′-UTRs of *AKT1* and *IKBKB*, leading to decreased NF-κB levels.^59^ We also found that LDEVs contain miRNA, and further analysis is currently underway (Figure S2).

Interestingly, we found that LDEVs have the potential for wound healing (Figure 4). It is well-known that wound healing is a complex physiological process, involving a series of inflammatory responses, cell proliferation, and cell migration. The healing process of skin wounds typically begins with an inflammatory response, which is the immediate reaction to skin damage. While inflammation helps to clear pathogens and damaged tissue from the wound area, paving the way for skin repair, excessive inflammation can negatively impact wound healing, delaying the repair process. Therefore, appropriate intervention with anti-inflammatory treatment can help mitigate this excessive response and accelerate wound healing. In addition, it can also reduce the pain and discomfort associated with inflammation.^60^ To our knowledge, only a few studies on PDEVs have focused on skin wound healing.^31^ Although lotus leaf possesses anti-inflammatory bioactivity, only one study has investigated the effects of lotus root on preventing skin photoaging.^61^ In the present study, our results indicate that appropriate concentrations of LDEVs can promote the proliferation and migration of human keratinocyte HaCaT cells (Figure 4). This paves the way for the application of LDEVs in wound healing. In the future, we will further investigate the underlying mechanisms.

## Conclusion

In this study, we evaluated the characteristics of LDEVs isolated by different methods and revealed the potential applications of LDEVs in anti-inflammatory and wound healing. By the use of a TFF system with a 750 kDa MWCO filter membrane, a higher yield of LDEVs with high purity can be effectively obtained. Surprisingly, although the LDEVs obtained by different methods had varying purities, this did not affect their anti-inflammatory activity, suggesting that the anti-inflammatory properties of LDEVs may be independent of the LDEV subtype. This provides new insights into whether PDEVs’ purity influences biological activity. The isolation method can also be applied to the separation of EVs from other plants, increasing the yield of PDEVs to accelerate the development of this field.

## Additional Experimental Details, Materials, and Methods

### RNA Extraction of Lotus-Derived EVs

Total RNA extraction was performed using TRIzol Reagent (Invitrogen, USA) according to the user guide. Briefly, 0.2 mL of LDEV solution was mixed with TRIzol reagent and incubated for 5 min, and 0.2 mL of chloroform was added. The tube was securely capped and thoroughly mixed by a vortex. After another 5 min incubation, the sample was centrifuged at 12,000*g* for 15 min at 4 °C. The aqueous layer containing RNA was carefully transferred to a new tube, and 0.5 mL of isopropanol was added to precipitate RNA. After incubation at −20 °C for 10 min, the sample was centrifuged at 12,000*g* for 10 min at 4 °C. The RNA pellet was washed twice with 75% ethanol and then resuspended in nuclease-free water (Invitrogen). The concentration and purity of the RNA sample were evaluated using a NanoDrop spectrophotometer (Thermo Fisher Scientific, US). RNA electrophoresis was performed using an Agilent 2100 Bioanalyzer (Agilent, US).

## Supplementary Material



## Notes

Schematic diagram of lotus-derive nanovesicles (LDEVs) after sucrose DGU and elution from qEV column and electropherogram of total RNA extracted from lotus-derived EVs (PDF)
