# Supplementary material for: Optimizing Isolation Methods and Exploring the Therapeutic Potential of Lotus-Derived Extracellular Vesicles in Modulating Inflammation and Promoting Wound Healing
Source: ACS Biomater Sci Eng. 2025 Jun 9;11(7):4424–35. doi: 10.1021/acsbiomaterials.5c00377 (PMC12264863; doi:10.1021/acsbiomaterials.5c00377)
Supplement: Supplementary file 1 [file ab5c00377_si_001.pdf]

Assessment of Isolation Methods and Revealing the Potential Application of Lotus-derived Extracellular Vesicles in Modulating Inflammation and Promoting Wound Healing

Kai-Jiun Lo[1], *Mu-Hui Wang*[2], *Ching-Yao Kuo*[3], *Min-Hsiung Pan*[1,4*]

[1] Institute of Food Science and Technology, National Taiwan University, Taipei 10617, Taiwan

[2] Department of Medical Research, National Taiwan University Hospital, Taipei 100225, Taiwan

[3] BO HUI BIOTECH CO., LTD., New Taipei City, Taiwan

[4] Department of Medical Research, China Medical University Hospital, China Medical University, Taichung 40402, Taiwan

*Correspondence: Min-Hsiung Pan; Email: mhpan@ntu.edu.tw

Material and Methods

RNA extraction and of Lotus-derived Extracellular Vesicles

Total RNA extraction was performed using TRIzol™ Reagent (Invitrogen, US) according to the user guide. Briefly, 0.2 mL of LDEVs solution was mixed with TRIzol reagent, incubated for 5 minutes, and 0.2 mL of chloroform was added. The tube was securely capped and thoroughly mixed by vortex. After another 5-minute incubation, the sample was centrifuged at 12,000 × g for 15 minutes at 4°C. The aqueous layer containing RNA was carefully transferred to a new tube, and 0.5 mL of isopropanol was added to precipitate RNA. After incubation at -20°C for 10 minutes, the sample was centrifuged at 12,000 × g for 10 minutes at 4°C. The RNA pellet was washed twice with 75% ethanol and then resuspended in nuclease-free water (Invitrogen). The concentration and purity of the RNA sample were evaluated using a NanoDrop spectrophotometer (Thermo Fisher Scientific, US). RNA electrophoresis was performed using the Agilent 2100 Bioanalyzer (Agilent, US).

Supplementary figures

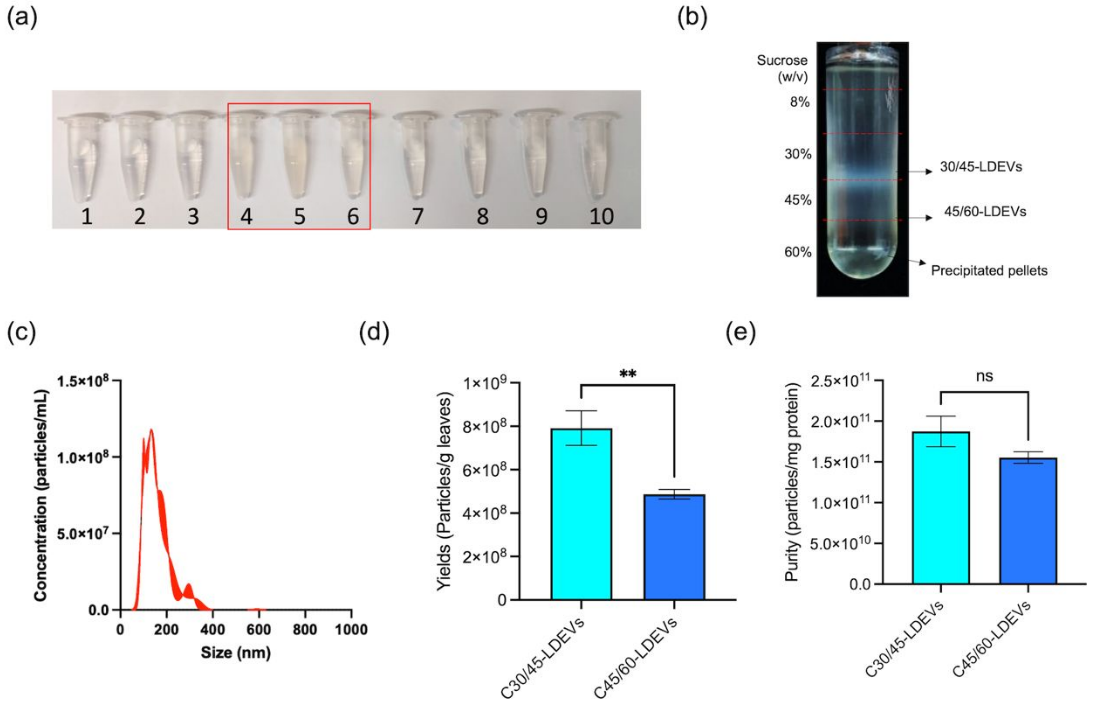

Figure S1. Schematic diagram of Lotus-derive nanovesicles (LDEVs) after sucrose density gradient ultracentrifugation and elution from qEV column. (a) Eluted fractions of LDEVs isolated through qEV column, with fractions 4-6 integrated as SEC-LDEVs. (b) 30/45- and 45/60-LDEVs were obtained from the interfaces between 30-45% and 45-60% sucrose gradient solutions, respectively. (c) Size distribution of precipitated pellets from the bottom of the centrifuged tube. (d) The yield of LDEVs isolated by integrated SEC-DGU strategy (e) Quantification of LDEV purification from integrated SEC-DGU strategy isolate LDEVs. Values are means ± SD and statistical analyses were conducted using *t*-test analysis. The data labeled with "*" indicate significant differences (p value < 0.05). ns: no significance.

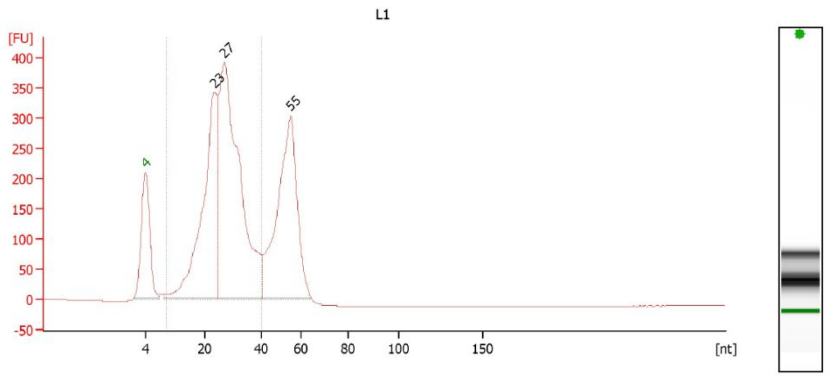

Figure S2. Electropherogram of total RNA extracted from lotus-derived extracellular vesicles. The peak at 4 nt represents a 4-nucleotide length standard. Peaks located around 20–40 nt represent the signals of miRNA.
